# TXNIP aggravates cardiac fibrosis and dysfunction after myocardial infarction in mice by enhancing the TGFB1/Smad3 pathway and promoting NLRP3 inflammasome activation: TXNIP aggravates MI-induced cardiac remodelling

**DOI:** 10.3724/abbs.2023150

**Published:** 2023-10-17

**Authors:** Yan Zhang, Jin Wang, Xuejiao Wang, Aiyun Li, Zhandong Lei, Dongxue Li, Dehai Xing, Yichao Zhang, Wanzhen Su, Xiangying Jiao

**Affiliations:** 1 Key Laboratory of Cellular Physiology (Shanxi Medical University) Ministry of Education and Department of Physiology Shanxi Medical University Taiyuan 030001 China; 2 Department of Foreign Languages Changzhi Medical College Changzhi 046000 China

**Keywords:** cardiac fibrosis, cardiac function, inflammasome, myocardial infarction, thioredoxin-interacting protein

## Abstract

Myocardial infarction (MI) results in high mortality. The size of fibrotic scar tissue following MI is an independent predictor of MI outcomes. Thioredoxin-interacting protein (TXNIP) is involved in various fibrotic diseases. Its role in post-MI cardiac fibrosis, however, remains poorly understood. In the present study, we investigate the biological role of TXNIP in post-MI cardiac fibrosis and the underlying mechanism using mouse MI models of the wild-type (WT), *Txnip*-knockout ( *Txnip*-KO) type and *Txnip*-knock-in ( *Txnip*-KI) type. After MI, the animals present with significantly upregulated TXNIP levels, and their fibrotic areas are remarkably expanded with noticeably impaired cardiac function. These changes are further aggravated under *Txnip*-KI conditions but are ameliorated in *Txnip*-KO animals. MI also leads to increased protein levels of the fibrosis indices Collagen I, Collagen III, actin alpha 2 (ACTA2), and connective tissue growth factor (CTGF). The *Txnip*-KI group exhibits the highest levels of these proteins, while the lowest levels are observed in the *Txnip*-KO mice. Furthermore, *Txnip*-KI significantly upregulates the levels of transforming growth factor (TGF)B1, p-Smad3, NOD-, LRR- and pyrin domain-containing protein 3 (NLRP3), Cleaved Caspase-1, and interleukin (IL)1B after MI, but these effects are markedly offset by *Txnip*-KO. In addition, after MI, the Smad7 level significantly decreases, particularly in the *Txnip*-KI mice. TXNIP may aggravate the progression of post-MI fibrosis and cardiac dysfunction by activating the NLRP3 inflammasome, followed by IL1B generation and then the enhancement of the TGFB1/Smad3 pathway. As such, TXNIP might serve as a novel potential therapeutic target for the treatment of post-MI cardiac fibrosis.

## Introduction

Cardiovascular diseases (CVDs) accounted for approximately one-third of global deaths in 2019 [1]. Among various CVDs, ischemic heart disease (IHD) is the leading condition, accounting for as much as 49.2% of the reported death toll [2]. Myocardial infarction (MI) refers to the death of a section of the myocardium, which is a consequence of the obstruction of coronary arteries [3]. As a major type of IHD, MI has a high incidence, which may result in a high hospitalization rate and high mortality, despite that great advances have been achieved in its treatment [ 4‒ 6]. Adverse post-MI cardiac remodeling contributes to the poor clinical outcomes of MI [7], which is manifested by fibrotic changes in the ventricular wall, leading to scar formation. Although scar size following MI is an independent predictor of cardiovascular outcomes [8], little is known about the influential factors for scar size. Therefore, how to control or even reverse adverse cardiac remodeling, particularly cardiac fibrosis, has become a prevention and treatment goal for MI.

Fibrosis is characterized by collagen deposition and extracellular matrix (ECM) formation [9]. Among various fibrosis-associated pathways, the transforming growth factor (TGF)B1/Smad2/3 signaling pathway has been extensively studied [ 10‒ 12]. In the process of canonical TGFB1 pathway signaling, TGFB1 pathway is activated by the phosphorylation of receptor-regulated (R)-Smads, while inhibitory (I)-Smads, induced by TGFB superfamily ligands, bind to type I receptors to prevent R-Smad phosphorylation, thereby inhibiting TGFB signaling [ 13 , 14]. Phosphorylation of R-Smads leads to their translocation to the nucleus, which induces the transcription of fibrosis-related genes [15]. Smad7 (an I-Smad), on the other hand, interacts physically with TGFB receptors and suppresses TGFB activation [16]. It is noteworthy that TGFB1 has been confirmed to be elevated after MI by numerous studies [ 17, 18]. Therefore, inhibiting TGFB1 signaling may offer potential for antifibrotic therapies.

The post-MI inflammatory reaction is not only a prerequisite for healing but also participates in scar formation [19]. Inflammasomes, intracellular multiprotein complexes, are able to distinguish between pathogen-associated molecular patterns (PAMPs) and damage-associated molecular patterns (DAMPs), which then trigger an inflammatory response in the host [20]. Among the main inflammasomes, NOD-, LRR- and pyrin domain-containing protein 3 (NLRP3) is one of the best-characterized inflammasomes [21]. PAMPs and DAMPs oligomerize and activate NLRP3 and recruit the adapter molecule, an apoptosis-associated speck-like protein that contains a C-terminal caspase-recruitment domain (CARD) (ASC) and pro-Caspase-1, to form a large complex. Afterwards, pro-Caspase-1 undergoes autoproteolytic cleavage to produce Caspase-1 and then activates pro-IL1B and pro-IL18 to produce the active proinflammatory cytokines IL1B and IL18 [22]. IL1B and IL18 exert biological functions by binding to IL1/18 receptors. Increasing evidence has suggested that fibrotic disease can be mediated by NLRP3 inflammasome signaling via IL1B, and IL1B promotes fibrogenesis via TGFB, ERK1/2, c-Jun, and PI3K/AKT [23]. However, whether NLRP3 inflammasome activation participates in post-MI cardiac fibrosis needs to be verified.

Thioredoxin-interacting protein (TXNIP), also named vitamin D3-upregulated protein 1 (VDUP1) or thioredoxin-binding protein 2 (TBP2), is a multifunctional protein involved in a variety of cellular processes, such as metabolism, oxidative stress, apoptosis, proliferation and inflammation [24]. Previous studies have shown that the expression of TXNIP increases in fibrotic diseases such as pulmonary fibrosis [25] and renal fibrosis [26]. Nevertheless, the role of TXNIP in post-MI cardiac fibrosis remains unclear. Given that the necrosis of myocardial cells after MI results in inflammation, which in turn worsens post-MI fibrosis [27] and that TXNIP can interact with NLRP3 [28], it is reasonable to hypothesize that TXNIP may play a role in these processes.

Therefore, in the present study, an MI-induced fibrotic mouse model with either *Txnip* gene knockout or knock-in was established to explore the role of TXNIP in post-MI cardiac fibrosis and the related underlying mechanisms. The results of this study might provide insight into clinical treatment aimed at restricting post-MI cardiac fibrotic progression.

## Materials and Methods

### Experimental animals and design

The animals used in this study included male C57BL/6J background homozygous *Txnip* knockout ( *Txnip*-KO) or *Txnip* knock-in ( *Txnip*-KI) mice and their wild-type (WT) littermates. The *Txnip*-KO and *Txnip*-KI mice were generated by GemPharmatech Co., Ltd. (Nanjing, China) using CRISPR/Cas9 technology that targeted the *Txnip* transcript ENSMUST00000074519.12. Specifically, *Txnip*-KO mice were created by knocking out exons 1‒8, whereas *Txnip*-KI mice experienced an insertion of a fragment of CAG-LSL- *Txnip*-P2A-EGFP-PolyA at the H11 site ( Supplementary Figure S1). All mice were bred and reared in a specific pathogen-free (SPF) environment under standardized conditions ( *i*. *e*., a fixed 12/12 h light/dark cycle and temperature of 22±2°C). They had free access to food and water. All animal experiments were conducted according to the guidelines on the Management of Experimental Animals issued by Shanxi Medical University.

When the mice weighed 22‒25 g, they were randomly divided into six groups as follows: WT-Sham, WT-MI, KO-Sham, KO-MI, KI-Sham and KI-MI groups.

### Establishment of the MI model

After the mice were verified for their specific genotype ( Supplementary Figure S2 and Table 1), those in the three MI groups were subjected to permanent left anterior descending (LAD) coronary artery ligation according to the method described in our previous work [29]. A marked pale color change in the ischemic area and an elevated ST segment on the electrocardiogram (ECG) indicated successful MI modeling. Mice without a noticeable elevated ST segment or those died after modeling were not included.

**Table:** **Table 1** Sequences of primers used for PCR

Primer	Primer sequence (5′→3′)	Length of products
KO	Forward	AAGAGGAGTCCCCTGGATGAGGTT	262 bp
Reverse	AAGACAACGCCAGAAGGTCAGC
KO-WT	Forward	CGCAACTTTCTGTCCAAGAAAGTG	407 bp
Reverse	ATCGAGAAAAGCCTTCACCCAGT
KI-3′	Forward	CCTGCTGTCCATTCCTTATTCCATA	329 bp
Reverse	ATATCCCCTTGTTCCCTTTCTGC
KI-5′	Forward	GGGCAGTCTGGTACTTCCAAGCT	335 bp
Reverse	TGGCGTTACTATGGGAACATACGTC
KI-WT	Forward	CAGCAAAACCTGGCTGTGGATC	412 bp
Reverse	ATGAGCCACCATGTGGGTGTC

Twenty-eight days after operation, cardiac function data were collected under isoflurane anesthesia. The mice were sacrificed, and their heart tissues were collected for subsequent experiments.

### Global cardiac function by ECG

At the end of the observation period, the mice were subjected to inhalation of 1.5% isoflurane, and transthoracic echocardiography was then performed at the papillary muscle level along the short axis with a 15 s MHz linear transducer. Parameters denoting cardiac function [ *i*. *e*., left ventricular ejection fraction (LVEF) and left ventricular fractional shortening (LVFS)] and those denoting heart structure [ *i*. *e*., left ventricular internal diameter at end systole (LVIDs), left ventricular internal diameter at end diastole (LVIDd), left ventricular anterior wall thickness at end systole (LVAWs), left ventricular anterior wall thickness at end diastole (LVAWd), left ventricular posterior wall thickness at end systole (LVPWs), and left ventricular posterior wall thickness at end diastole (LVPWd)] were measured with a two-dimensional guided M-mode ultrasound system (Vivid 7; GE Healthcare, Waukesha, USA). Two other indicators were also calculated, including left ventricular end systolic volume (LVESV) and left ventricular end diastolic volume (LVEDV).

### Heart weight/body weight (HW/BW) ratio determination and heart sample preparation

At the end of the experiment, the mouse heart was removed and cleaned in ice-cold saline. After drying, the heart HW/BW (mg/g) ratio was calculated. The heart tissue was cut below the ligation along the transverse section and then divided into two parts by cutting along the transverse section approximately 0.3 mm below, with the upper part paraffin-embedded and the lower part immediately frozen in liquid nitrogen. The paraffin-embedded sample was sliced into 5-μm sections with a Leica RM2245-Semi Motorized Rotatory Microtome (Leica, Nussloch, Germany).

### Masson’s trichrome staining and picrosirius red staining

Masson’s trichrome staining and picrosirius red staining were performed to evaluate interstitial collagen deposition in the cardiac tissue at 28 days after the operation, and quantitative analysis was conducted using morphometry. Sections from the mid-ventricular level from four mice in each group were subject to Masson staining according to the manufacturer’s instructions (D026-1-3; Nanjing Jiancheng Bioengineering Institute, Nanjing, China). Picrosirius red staining (Anhui Leagene Biotechnology, Co., Ltd, Huaibei, China) was performed to observe the infarct size using light microscopy and Collagen I and III contents using polarized light microscopy. Images were taken by using a Nikon Ni-SS upright microscope (Nikon ECLIPSE Ti2; Nikon Corporation, Tokyo, Japan) and a polarized light microscope (Panthera I; Motic China Group Co., Ltd, Xiamen, China).

### Immunohistochemical staining

The prepared cardiac tissue sections were deparaffinized, rehydrated and then subjected to a 20-min heat-mediated (95°C) antigen retrieval in 10 mM sodium citrate buffer (pH 6.0) for actin alpha 2 (ACTA2) and in Tris-EDTA buffer (pH 9.0) for Collagens I and III. After cooling to room temperature (RT), the sections were incubated in 3% H _2_O _2_ for 10 min to block endogenous peroxidase followed by blocking in 5% serum for 1 h. The sections were incubated with primary antibodies: monoclonal rabbit anti-Collagen I (ab270993, 1:500 dilution; Abcam, Cambridge, UK); and monoclonal rabbit anti-alpha smooth muscle actin antibody (ab124964, 1:2000 dilution; Abcam) at 4°C overnight, followed by incubation with a secondary antibody (goat anti-rabbit IgG/HRP; PV-9001; ZSGB-BIO, Beijing, China) for 20 min at RT. For Collagen III, the primary antibody was mouse monoclonal antibody (M00788; Boster, Wuhan, China) and the incubation condition was 37°C for 30 min. The secondary antibody was biotin-labeled goat anti-mouse IgG and Strept Avidin-Biotin Complex (SABC) solution from the SABC immunohistochemical staining kit (SA1021; Boster). The 3,3′-diaminobenzidine (DAB) chromogenic kit (ZLI-9018; ZSGB-BIO) was used as the chromogen, and the immunolabelled sections were counterstained with hematoxylin. Finally, each section was imaged for the peri-infarct area under three different high-power fields with an Olympus BX53 microscope (BX53F; Olympus, Tokyo, Japan), and the deposition area was calculated by using ImageJ software (v.1.42; NIH, Bethesda, USA).

### Quantitative real-time polymerase chain reaction

Total mRNA was extracted from the myocardial tissue using RNAiso plus (TaKaRa, Kyoto, Japan). NanoDrop One (Thermo Scientific, Waltham, USA) was used to detect the concentration and purity of the extracted mRNA, and the PrimeScrip RT kit (TaKaRa) was used for reverse transcription of total RNA to cDNA. This reaction was conducted in a LightCycler 96 real-time PCR system (Roche, Basel, Switzerland). The final amplification reaction volume was 20 μL, and the conditions were 95°C for 30 s in preincubation and 45 cycles of 95°C for 10 s, 60°C for 10 s, and 72°C for 10 s, followed by the final melting and cooling processes. The sequences of the primers used are shown in Table 2. Relative quantification was calculated using the 2 ^‒ΔΔCT^ method. *GAPDH* was used as the internal reference for gene level detection.

**Table:** **Table 2** Sequences of primers used for qPCR

Gene	Primer sequence (5′→3′)
*Txnip*	Forward	CAAGGGTCTCAGCAGTGCAAAC
Reverse	AAGCTCGAAGCCGAACTTGTACTC
*GAPDH*	Forward	TGTGTCCGTCGTGGATCTGA
Reverse	TTGCTGTTGAAGTCGCAGGAG

### Western blot analysis

The protein levels of Collagen I, Collagen III, ACTA2, CTGF, TXNIP, TGFB1, Smad3, p-Smad3, Smad7, NLRP3, ASC, Caspase-1, Cleaved Caspase-1, IL1B, and GAPDH were determined by western blot analysis. Briefly, heart tissue samples from different groups were homogenized in RIPA lysis buffer containing 1% protease inhibitor (AR1179; Boster) and 1% phosphatase inhibitors (AR1183; Boster). The protein concentration was determined using the BCA protein assay reagent kit (AR1189; Boster). Then, the protein lysate was boiled and loaded onto gels for SDS-PAGE. The protein was transferred from the gel onto a polyvinylidene fluoride membrane (Millipore, Billerica, USA). Then, the membrane was incubated with one of the following antibodies at 4°C overnight: rabbit anti-Collagen I, ACTA2, CTGF, TXNIP, Smad3, p-Smad3 (Abcam), anti-Smad7, NLRP3, ASC, Caspase-1/Cleaved Caspase-1, Mature IL1B, GAPDH (Wanleibio, Shenyang, China), mouse anti-TGFB1 antibody (Santa Cruz Biotechnology, Dallas, USA), and mouse anti-Collagen III antibody (Boster). Secondary antibodies from the corresponding sources were then used, and the antibody-antigen complexes on each membrane were detected using the Super ECL Prime kit (SEVEN Biotech, Beijing, China). Protein bands were exposed by the ChemiDocTM Touch imaging system (Bio-Rad Laboratories, Hercules, USA) and then analyzed based on the gray value using ImageJ software. GAPDH was used as the internal protein loading control.

### Statistical analysis

All data are presented as the mean±standard error of the mean (SEM). Statistical analysis was performed using GraphPad Prism 8 software (GraphPad Software, Inc., La Jolla, USA). Two-way analysis of variance (ANOVA) was used to analyze the differences between groups. *P*<0.05 was considered statistically significant.

## Results

### TXNIP level was increased after MI, and TXNIP exacerbated post-MI cardiac dysfunction

After LAD ligation, the apex and left ventricle (LV) anterior wall became ischemic, and the ST segment exhibited an elevation according to ECG, which indicated that an MI mouse model was successfully established ( Supplementary Figure S3).

**Figure 1:**
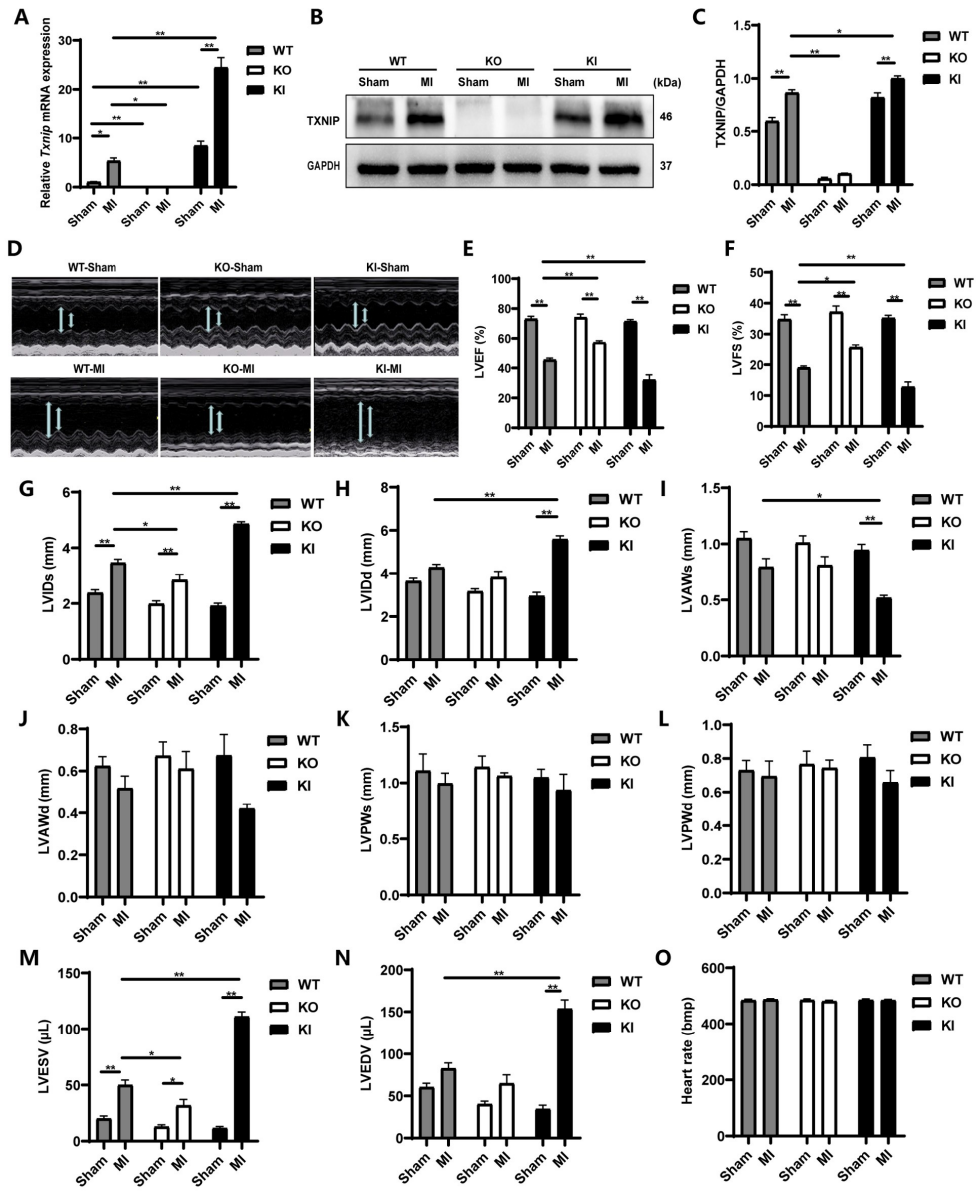
Figure 1 Increased expression of TXNIP after MI aggravated post-MI cardiac dysfunction (A) The Txnip mRNA level detected by qPCR shows an increase after MI. Data are shown as the mean±SEM, n=4. *P<0.05, **P<0.01. (B) Representative western blots of TXNIP. (C) The protein level of TXNIP detected by western blot analysis shows an increase after MI. Data are shown as the mean±SEM, n=4. *P<0.05, **P <0.01. (D) Representative echocardiographic images of each group at 28 days after MI. (E‒N) Statistical analyses of LVEF, LVFS, LVIDs, LVIDd, LVAWs, LVAWd, LVPWs, LVPWd, LVESV, and LVEDV. (O) Statistical analysis of heart rate. Data are shown as the mean±SEM, n=5/6. *P<0.05, **P<0.01. LVEF, left ventricular ejection fraction; LVFS, left ventricular fractional shortening; LVIDs, left ventricular internal diameter at systole; LVIDd, left ventricular internal diameter at diastole; LVAWs, left ventricular anterior wall thickness at systole; LVAWd, left ventricular anterior wall thickness at diastole; LVPWs, left ventricular posterior wall thickness at systole; LVPWd, left ventricular posterior wall thickness at diastole; LVESV, left ventricular end-systolic volume; and LVEDV, left ventricular end-diastolic volume.

Then, the mRNA and protein levels of cardiac TXNIP were determined. After MI, the mRNA and protein levels of cardiac TXNIP in both the WT and *Txnip*-KI groups were significantly upregulated compared with their corresponding sham groups ( Figure 1A‒C).

Cardiac function was evaluated using ECG 28 days after the operation ( Figure 1D‒N). Parameters associated with overall LV systolic function, *i*. *e*., LVEF and LVFS, showed significant decreases in the three MI groups compared with their corresponding sham groups ( *P*<0.01). Although LVAWs and LVAWd were decreased in the three post-MI mice, a significant difference was observed only in LVAWs between the KI-Sham and KI-MI groups ( *P*<0.01). Among the MI groups, the lowest LVEF, LVFS, and LVAWs were observed in the KI-MI mice, while the highest were observed in the KO-MI mice, all showing statistically significant differences compared with those in the WT-MI group ( *P*<0.05, *P*<0.01), except for the LVAWs between the WT-MI group and the KO-MI group. The remaining indicators, including LVIDs, LVIDd, LVESV and LVEDV, all increased in the MI groups compared with their corresponding sham groups, and significant differences were observed in all four indices between the WT-MI and KI-MI groups, while the WT-MI group and the KO-MI group only showed significant differences in LVAWs and LVESV ( *P* <0.05, *P*<0.01). Additionally, no difference in heart rate was observed among the groups, despite a slight increase after MI ( Figure 1O). These findings indicated that cardiac function was significantly compromised by *Txnip* overexpression.

### TXNIP increased the infarct size after MI

**Figure 2:**
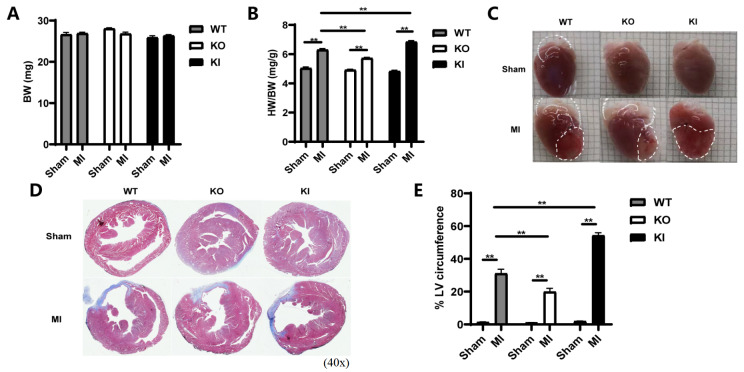
Figure 2 TXNIP increased the post-MI infarct size (A) The BW of the mice in each group. Data are shown as the mean±SEM, n=6. (B) The HW/BW ratio of the mice in each group. Data are shown as the mean±SEM, n=6. **P<0.01. (C) Representative gross view images of the heart from each group at 28 days after treatment. (D) Representative images of the heart from each group at 28 days after treatment, after Masson staining. (E) Infarct size presented as the percentage of the LV circumference at the mid-ventricular level. Data are shown as the mean±SEM, n=4. BW, body weight; HW, heart weight; HW/BW ratio, the ratio of HW to BW.

The body weight (BW) of each mouse was obtained 28 days after the operation, and no significant difference was observed among the groups ( Figure 2A). After animal sacrifice, the ratio between the heart weight (HW) and the body weight (HW/BW) was calculated. After MI, the HW/BW ratio was increased. Among the three MI groups, the lowest HW/BW ratio was observed in the KO-MI group, whereas the highest was observed in the KI-MI groups, and significant differences were observed among the three groups ( *P*<0.01; Figure 2B).

The gross view of the heart revealed that the pale infarcted area of the KI-MI group was larger than that of the WT-MI counterpart, while the KO-MI group displayed a smaller infarcted area ( Figure 2 C).

**Figure 3:**
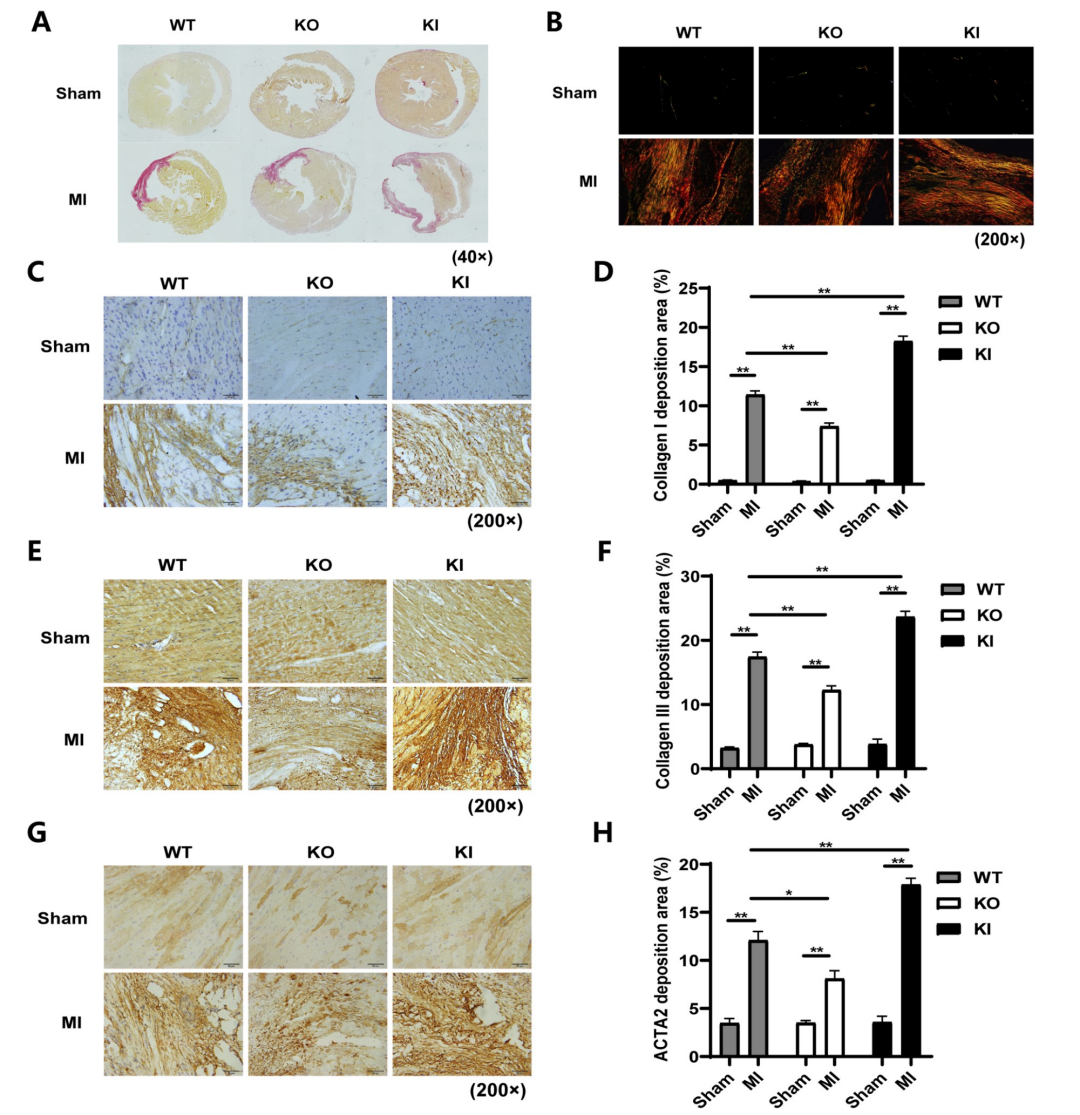
Figure 3 TXNIP promoted post-MI collagen deposition (A,B) Representative picrosirius red staining images of heart sections from each group at 28 days after treatment (A) under a light microscope and (B) under a polarized light microscope. Collagen I showed red/orange birefringence, while Collagen III showed green/whitish birefringence. (C,E,G) Representative immunohistochemical staining images of heart sections from each group at 28 days after treatment. (C) Collagen I deposition. (E) Collagen III deposition, and (G) ACTA2 deposition. (D) Statistical analysis of Collagen I deposition. Data are shown as the mean±SEM, n=3. **P<0.01. (F) Statistical analysis of Collagen III deposition. Data are shown as the mean± SEM, n=3. **P<0.01. (H) Statistical analysis of ACTA2 deposition. Data are shown as the mean±SEM, n=3. *P<0.05, **P<0.01.

The infarct size was further determined by Masson staining, which was presented as the percentage of the LV circumference at the mid-ventricular level [30]. Collagen was stained blue, viable muscle fibres appeared purple red, and the nuclei turned black. Collagen content was detected using polarized light microscopy for the sections stained with picrosirius red; Collagen I exhibited red/orange birefringence, and Collagen III displayed green/whitish birefringence. The results revealed that the infarct size became significantly larger after MI ( *P* <0.01; Figure 2D and Figure 3A,B). Compared with the WT-MI mice, the KO-MI mice presented with a significantly reduced infarct size, while the KI-MI mice showed a significantly increased infarct size ( *P*<0.01; Figure 2E).

All these findings indicated that *Txnip*-KI exacerbated cardiac fibrotic remodeling and increased the infarct size after MI, whereas *Txnip*-KO played a protective role in cardiac fibrotic remodeling after MI and reduced the infarct size.

### TXNIP increased the protein levels of Collagen I, Collagen III, ACTA2 and CTGF after MI

**Figure 4:**
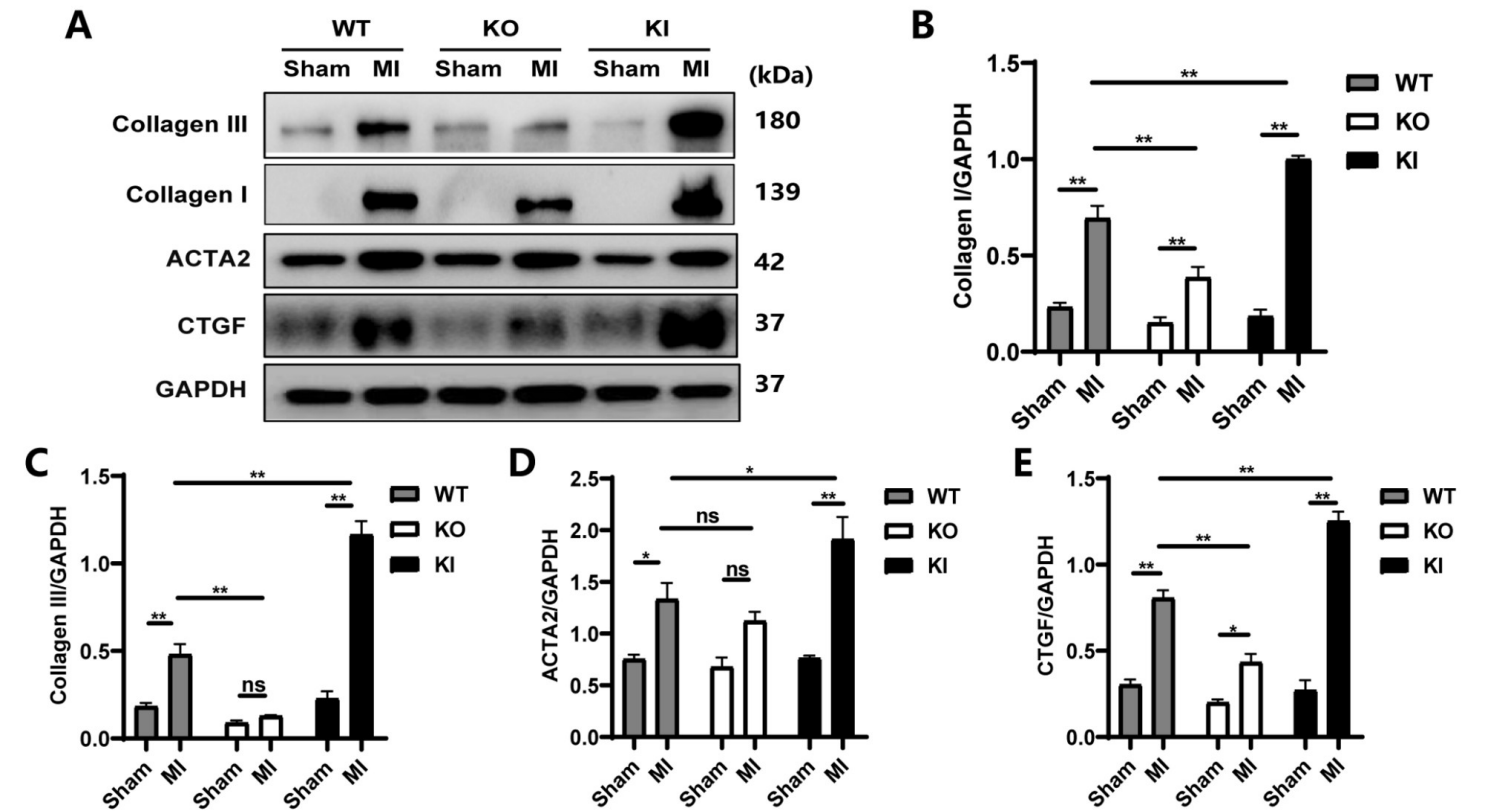
Figure 4 TXNIP increased post-MI collagen deposition (A) Representative images of the protein levels of Collagen III, Collagen I, ACTA2, and CTGF. (B‒E) Statistical analyses of the protein levels of Collagen I, Collagen III, ACTA2, and CTGF. Data are shown as the mean±SEM, n=3‒5. *P<0.05, **P<0.01; ns, not significant.

The levels of Collagen I, Collagen III, ACTA2, and CTGF were all elevated in the MI groups compared with their corresponding sham groups ( *P* <0.05, *P*<0.01), except those of Collagen III and ACTA2 between the KO-Sham group and the KO-MI group. Compared with the WT-MI group, the KI-MI group showed significantly increased expressions of Collagen I, Collagen III, and CTGF ( *P*<0.01), while the KO-MI group showed a significant decrease in these indices ( *P*<0.01; Figure 3C‒F and Figure 4A‒C,E). The results based on western blot analysis showed slight differences in the ACTA2 level, except for significantly higher expression in the KI group than that in the WT group after MI ( *P* <0.05; Figure 4D). These results are somewhat different from those of immunohistochemical staining, according to which ACTA2 also showed a significant difference between the KO-Sham group and the KO-MI group ( *P*<0.01; Figure 3G,H).

The findings here suggested that *Txnip*-KO helped reduce collagen deposition after MI, thereby improving cardiac compliance and finally ameliorating cardiac dysfunction; however, *Txnip*-KI worsened post-MI cardiac fibrosis, resulting in more compromised cardiac function.

### TXNIP aggravated post-MI collagen deposition by enhancing the TGFB1/Smad3 pathway

**Figure 5:**
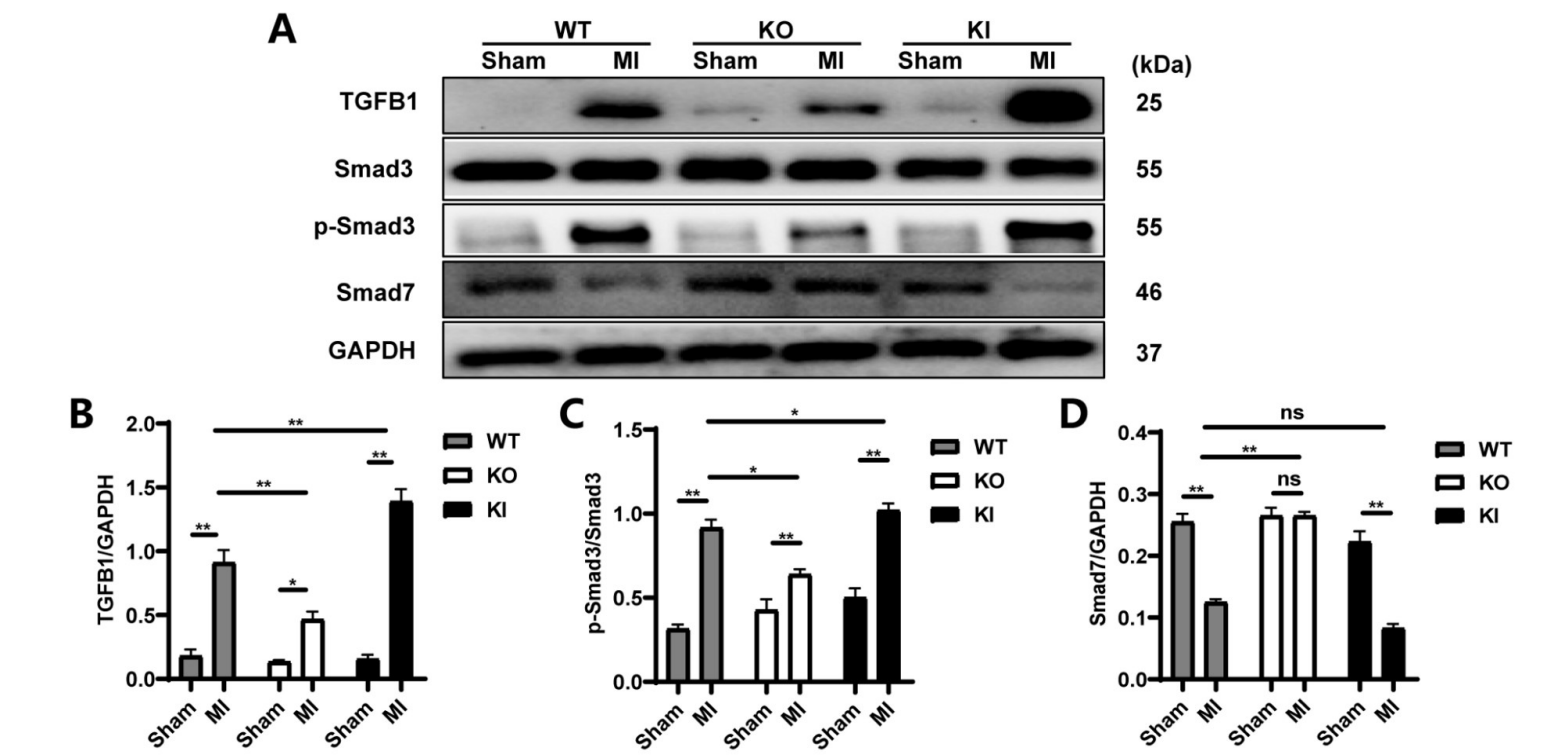
Figure 5 TXNIP enhanced the TGFB1/Smad3 pathway and decreased Smad7 levels (A) Representative images of the protein levels of TGFB1, Smad3, p-Smad3, and Smad7. (B‒D) Statistical analysis of the protein levels of TGFB1, p-Smad3/Smad3 ratio, and Smad7. Data are shown as the mean±SEM, n=4. *P<0.05, ** P<0.01; ns, not significant.

We next explored the possible mechanisms underlying the relationship between post-MI deposition of collagen and TXNIP. The TGFB1/Smad3 pathway is a well-known signaling pathway that influences fibrogenesis. We found that MI resulted in significantly increased expression of TGFB1 and an elevated p-Smad3/Smad3 protein ratio but a decreased Smad7 level in all three types of mice ( *P*<0.05, *P*<0.01; Figure 5). This finding might be correlated with the increased expressions of Collagens I and III and CTGF we previously found. Furthermore, the expression of TGFB1 and the p-Smad3/Smad3 ratio were significantly reduced in the KO-MI mice but significantly increased in the KI-MI mice compared with their WT-MI counterparts ( *P*<0.05; Figure 5). This finding suggested that *Txnip*-KO inhibited the activation of the TGFB1/Smad3 signaling pathway and therefore led to less severe collagen deposition. The opposite changes occurred under *Txnip*-KI conditions.

### TXNIP aggravated post-MI collagen deposition by enhancing the TGFB1/Smad3 pathway and promoting NLRP3 inflammasome activation

**Figure 6:**
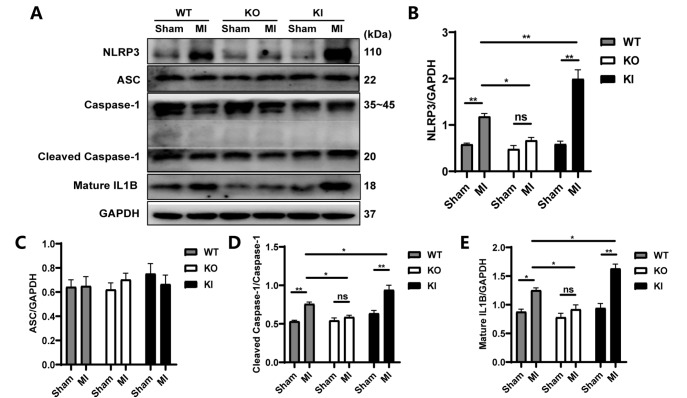
Figure 6 TXNIP promoted NLRP3 inflammasome activation (A) Representative images of the protein levels of NLRP3, ASC, Caspase-1, Cleaved Caspase-1, and Mature IL1B. (B‒E): Statistical analysis of the protein levels of NLRP3, ASC, Cleaved Caspase-1/Caspase-1, and Mature IL1B. Data are shown as the mean±SEM, n=4. *P<0.05, **P<0.01; ns, not significant.

Considering that TXNIP interacts directly with the NLRP3 inflammasome and that inflammation has established effects on fibrosis, we continued to investigate whether there are changes in the NLRP3 inflammasome and downstream biomarkers and whether a relationship between TXNIP and the NLRP3 inflammasome exists in our modeled mice. The levels of the key components of the NLRP3 inflammasome, including NLRP3 and Cleaved Caspase-1 (P20), were consistent with those of their upstream regulator TXNIP, showing significant upregulation after MI in the WT and KI mice ( *P*<0.01; Figure 6A,B,D). The protein level of the downstream proinflammatory cytokine IL1B was also significantly upregulated in the WT-MI and KI-MI groups ( *P* <0.05, *P*<0.01; Figure 6A,E). In addition, the levels of NLRP3, Cleaved Caspase-1/Caspase-1, and IL1B were the highest in the KI-MI mice but the lowest in the KO-MI mice among the three types of MI mice ( Figure 6A,B,D,E). Therefore, our study demonstrated that *Txnip*-KI promoted the inflammatory response by enhancing the TXNIP/NLRP3 inflammasome signaling cascade, while *Txnip*-KO suppressed the inflammatory response by inhibiting this cascade.

The results derived from collagen detection suggested that the reduced deposition of Collagen I and Collagen III after MI might be associated with the inhibitory effect of *Txnip*-KO on NLRP3 inflammasome activation and the subsequent reduced production of IL1B and less positive feedback on TGFB1/Smad3 signaling, while *Txnip* knock-in aggravated post-MI collagen deposition by enhancing the TGFB1/Smad3 pathway and promoting NLRP3 inflammasome activation.

## Discussion

MI is a major public health problem that leads to a great number of deaths each year, and post-MI cardiac fibrosis without timely intervention contributes to its high mortality. Therefore, exploration of new therapeutic targets and strategies for restricting or reducing post-MI fibrosis is urgent in the treatment of MI. In this study, we found that the TXNIP level was elevated after MI and that TXNIP aggravated post-MI fibrosis and cardiac dysfunction by enhancing NLRP3 inflammasome activation, promoting the generation of the inflammatory cytokine IL1B and enhancing the TGFB1/Smad3 pathway. These findings suggest that TXNIP might be a novel potential therapeutic target for the treatment of post-MI cardiac fibrosis.

TXNIP is a member of the α-arrestin protein family, with ubiquitous expression within the body. It is involved in the regulation of multiple cellular processes including inflammation. Post-MI cardiomyocyte necrosis induces an inflammatory reaction, which aggravates post-MI fibrosis [27]. However, the role of TXNIP in post-MI fibrosis remains unclear to date. Moreover, studies on TXNIP overexpression have rarely been reported. A recent study demonstrated the existence of ubiquitous TXNIP overexpression in CBA ^TXNIP/+^ mice, which aggravated the severity of streptozotocin-induced diabetes [31]; however, this study did not report the influence of TXNIP overexpression on cardiac injury. Considering that the majority of previous studies focusing on TXNIP involved the use of *Txnip*-KO animals or cells, we established the *Txnip*-KI mouse model to explore the role of TXNIP in post-MI fibrosis in a general TXNIP overexpression setting based on comparison of the results obtained from the WT, *Txnip*-KO, and *Txnip*-KI mice.

First, we successfully constructed *Txnip*-KO and *Txnip*-KI transgenic mice. Sex differences in the pathophysiology of cardiac fibrosis are evident [32], and female mice present with less extensive left ventricular remodeling after MI [33] because of the repairing function of estrogen [34]. To exclude the possibility of the protective role of estrogen in the heart, we only used male mice. TXNIP is a glucose regulator. Detection of the fasting blood glucose levels of the normal 8-week-old WT, KO, and KI mice showed that the KO mice had a significantly decreased blood glucose level, but KI mice had a significantly increased level compared with the WT mice ( Supplementary Figure S4). The cardiac function indices of each type of mouse showed no significant differences, which indicated that the slight difference in blood glucose among the different mouse groups was not the primary cause of cardiac left ventricular dysfunction. Then, we performed LAD ligation to induce MI, and sham operation on the WT and both types of *Txnip* genetically engineered mice was performed. We found that the level of TXNIP was elevated in the WT and KI heart tissues after MI compared with their corresponding sham groups. Since all eight exons of the *Txnip* gene were knocked out in the KO mice, no difference in the level of TXNIP was observed in the KO mice before and after MI. Echocardiography showed that cardiac function became poorer after MI, with the poorest performance observed in the *Txnip*-KI mice. A post-MI collagen-based scar is a noncontractile tissue that contributes to cardiac systolic and diastolic dysfunctions, although it maintains the integrity of the ventricle [35]. Our results obtained from Masson staining and picrosirius red staining were consistent with those reported in the literature. According to this study, the largest infarcted heart area was observed in the *Txnip*-KI mice after MI, which indicated that TXNIP might be a detrimental factor for post-MI fibrosis.

Then, we explored the content of collagen after MI in the heart because post-MI fibrosis is associated with collagen accumulation. Generally, Collagen I and Collagen III are predominantly responsible for interstitial fibrosis, accounting for more than 90% of the total collagen content of the heart [15]. Based on immunohistochemical staining and western blot analysis, we found that the levels of both Collagen I and Collagen III were increased after MI, with the highest amount in the KI-MI mice and the lowest amount in the KO-MI mice. In addition, the levels of ACTA2 and CTGF, which cause fibrogenesis and scar tissue in various pathological conditions, exhibited the same changes as those of Collagen I and Collagen III. Considering that these four indicators are all fibrosis-related proteins, the results in our study strongly indicated that TXNIP overexpression aggravated post-MI collagen deposition and fibrosis.

Because the genes encoding Collagens I/III, ACTA2, and CTGF are all downstream target genes of the TGFB1/Smad signaling pathway, we investigated the changes in signaling molecules in this pathway. We found that the level of TGFB1 was increased after MI. Upon binding of TGFB1 to its receptor, R-Smads, namely, Smad2 and Smad3, are phosphorylated and then translocated to the nucleus, where they act as transcription factors and induce the transcription of fibrosis-related genes [15]. We found that after MI, all three types of mice showed a significantly elevated p-Smad3/Smad3 ratio, where the most profound change was observed in the KI-MI mice, while the least increase was observed in the KO-MI mice. Nevertheless, no significant difference was observed in the pSmad2/Smad2 ratio among the three MI groups ( Supplementary Figure S5). Smad7, an I-Smad, binds stably to activated type I receptors; it competes with Smad3 for receptor activation [36] and negatively regulates the phosphorylation of Smad3. In this study, we found a significant decrease in Smad7 in the WT and KI mice after MI, with the lowest amount in KI-MI mice and the highest amount in KO-MI mice. The findings with regard to the TGFB1/Smad signaling pathway here partially support the increased expressions of Collagens I/III, ACTA2 and CTGF reported above.

We continued to explore the possible mechanism underlying the relationship between TXNIP and TGFB1. Inflammation-mediated cardiac fibrosis is one of the key mechanisms in the pathogenesis of heart failure. Although the post-MI inflammatory process is necessary for tissue healing, it may cause damage to the myocardium, leading to maladaptive ventricular remodeling and even cardiac dysfunction and heart failure [19]. Inflammasomes are sensors that initiate the inflammatory response within cells. The NLRP3 inflammasome became our focus since it can bind directly to TXNIP [28]. Blocking their interaction is able to alleviate hypertensive cardiac inflammation and fibrosis [37]. However, little is known about the role of TXNIP and NLRP3 in post-MI fibrosis. The NLRP3 inflammasome is a multiprotein complex that consists of NLRP3, ASC, and Caspase-1. ASC acts as the bridge between NLRP3 and Caspase-1, which contributes to inflammasome activation and the subsequent maturation and secretion of IL1B and IL18 [38]. Without stimulation, TXNIP is bound to and inhibited by thioredoxin. Following an increased level of cellular reactive oxygen species (ROS), *e*. *g*., after acute MI [39], the complex dissociates, and TXNIP binds to NLRP3, leading to NLRP3 activation [28]. Activation of the NLRP3 inflammasome amplifies the inflammatory response and mediates tissue damage [40]. After MI, the expression of NLRP3 is upregulated in the left ventricle, primarily in myocardial fibroblasts [41]. In our study, increased expression of heart NLRP3 was found after MI modeling in mice compared with sham mice, with the greatest increase observed in the *Txnip*-KI-MI mice and the least in the *Txnip*-KO-MI mice. Consistent with the change in the NLRP3 level, the levels of Cleaved Caspase-1 and IL1B showed the same trends.

TXNIP/NLRP3 inflammasome-mediated maturation of IL1B is involved in organ inflammation and fibrosis, which is known to constitute a positive feedback to TGFB1 activation [42]. In addition, IL1B is a potent inducer of TGFB [43]. In our study, under *Txnip-*KI conditions, the levels of TXNIP, NLRP3, Cleaved Caspase-1/Caspase-1, IL1B, and TGFB1 and the p-Smad3/Smad3 ratio showed the most significant increase after MI, and collagen deposition and scar size were also most noticeable among the three MI groups. However, these changes were reversed by *Txnip*-KO.

**Figure 7:**
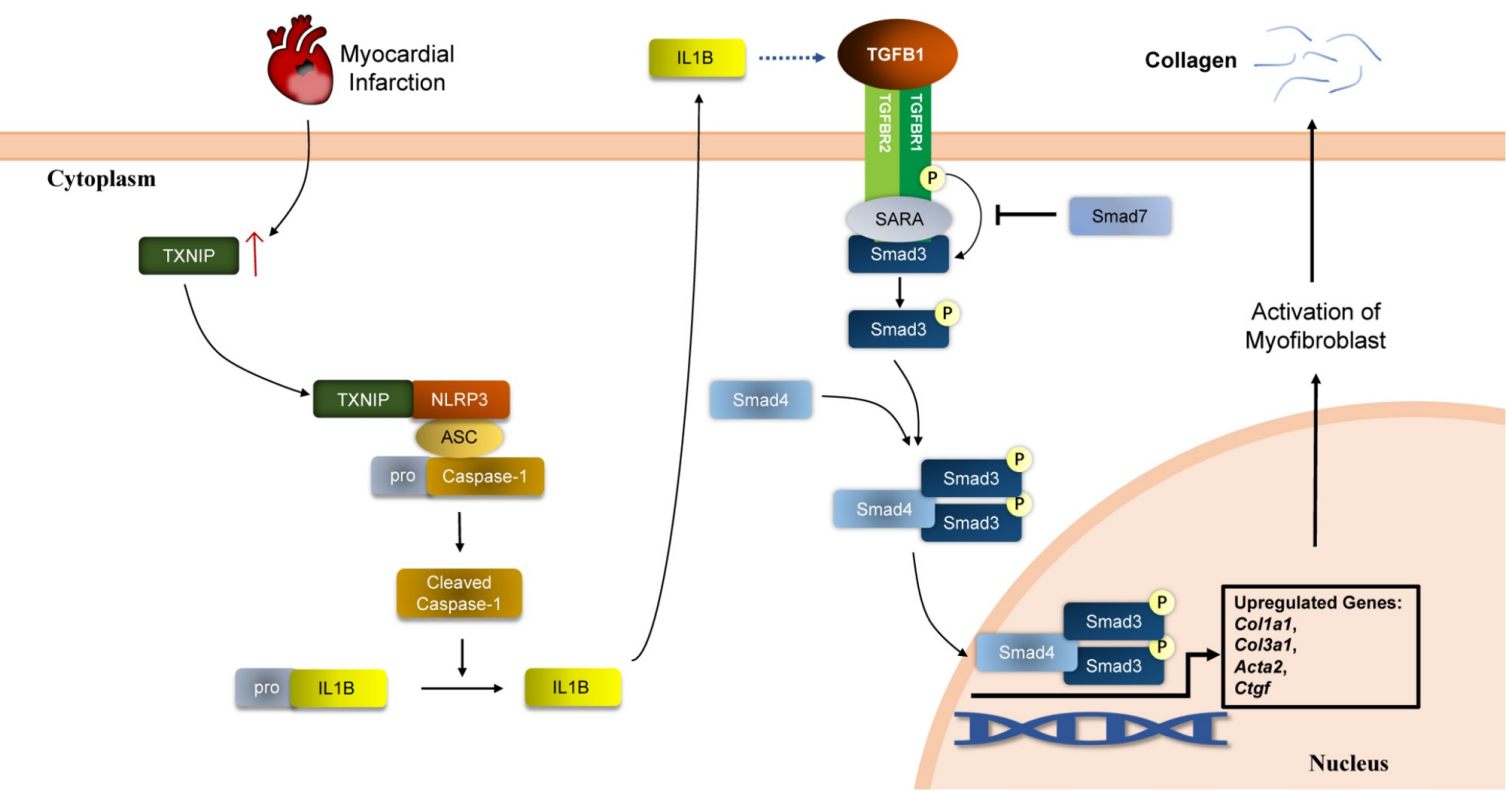
Figure 7 The proposed mechanism by which TXNIP aggravates post-MI cardiac fibrosis and dysfunction The increased level of TXNIP after MI promoted the activation of NLRP3 inflammasome. Subsequently, the production of mature IL1B, the downstream of NLRP3 was increased. After that, the TGFB1/Smad3 signaling pathway was also enhanced, leading to transcription of fibrosis-related genes and synthesis of collagen fibres. Therefore, we can conclude that TXNIP aggravates post-MI cardiac fibrosis and dysfunction by enhancing the TGFB1/Smad3 pathway and promoting NLRP3 inflammasome activation.

In summary, our results indicate that TXNIP plays a role in cardiac fibrotic remodeling after MI. It activates the NLRP3 inflammasome and promotes the release of the proinflammatory cytokine IL1B ( Figure 7). In this way, it enhances TGFB1/Smad3-mediated collagen deposition, promotes the formation of increased scar areas and eventually exacerbates cardiac dysfunction.

## Supplementary Data

Supplementary data is available at *Acta Biochimica et Biophysica Sinica* online.

## Supporting information

23223Supplementary_Figures
