# Supplementary material for: TXNIP aggravates cardiac fibrosis and dysfunction after myocardial infarction in mice by enhancing the TGFB1/Smad3 pathway and promoting NLRP3 inflammasome activation: TXNIP aggravates MI-induced cardiac remodelling
Source: Acta Biochim Biophys Sin (Shanghai). 2023 Oct 17;55(12):1950–60. doi: 10.3724/abbs.2023150 (PMC10753373; doi:10.3724/abbs.2023150)
Supplement: 23223Supplementary_Figures [file 23223Supplementary_Figures.pdf]

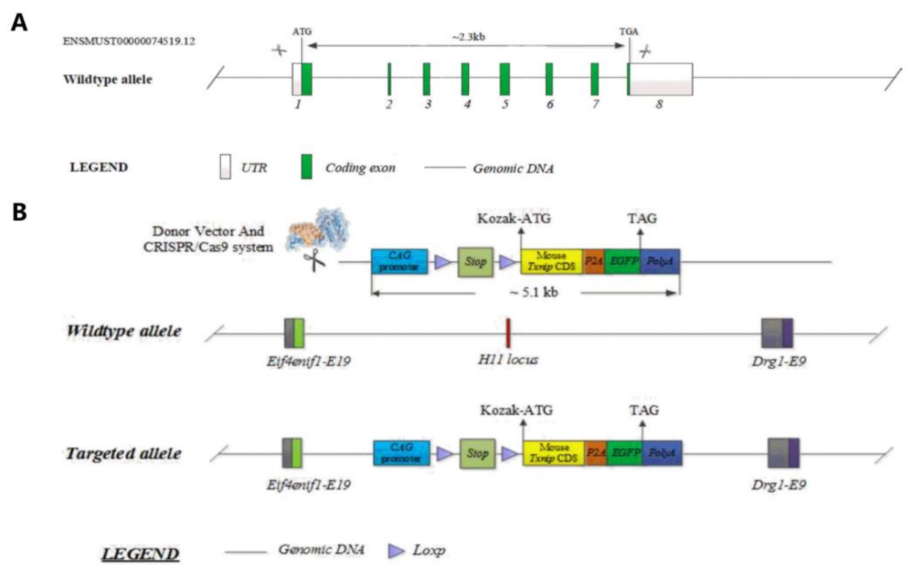

Supplementary Figure S1. Overview of the targeting strategy for the *Txnip*-KO and *Txnip*-KI mice (A) The targeting strategy for the *Txnip*-KO heterozygous mice. (B) The targeting strategy for the *Txnip*-KI heterozygous mice. *Txnip*, thioredoxin-interacting protein; KO, knockout; KI, knock-in.

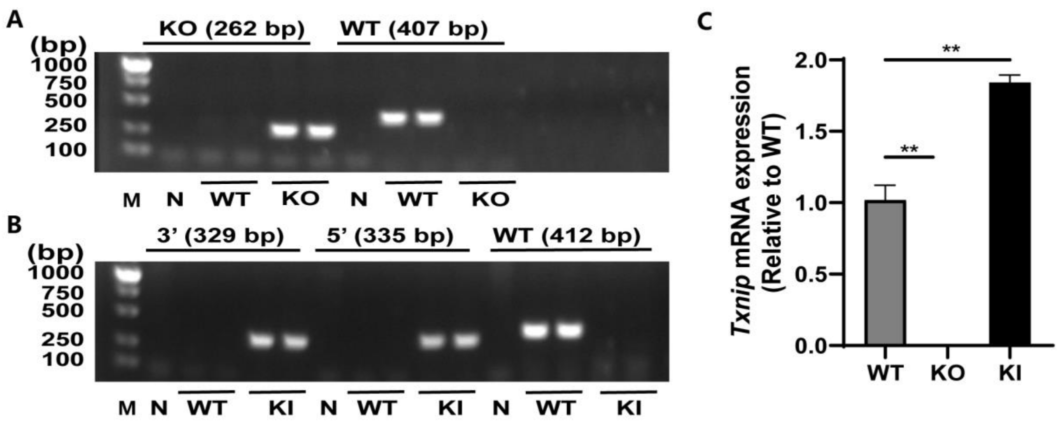

Supplementary Figure S2. Gene expression determined by PCR (A) *Txnip*-KO mice. (B) *Txnip*-KI mice. (C) The mRNA level of *Txnip* in the WT, KO, and KI mice. Data are shown as the mean ± SEM, n=5. **P<0.01. WT, wild type; M, DNA marker; N, negative control.

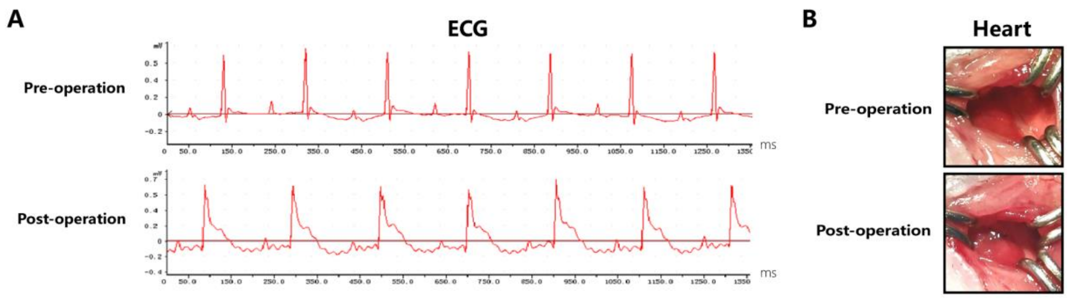

Supplementary Figure S3. Successful establishment of the mouse MI model (A) ECG displayed an elevated ST segment after MI. (B) The ventricular wall of the heart became ischemic after MI.

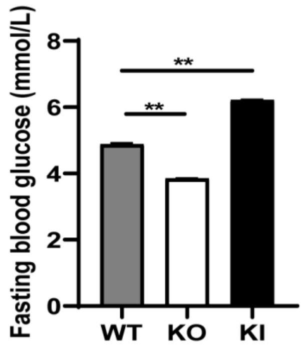

Supplementary Figure S4. Blood glucose levels of the three types of mice Data are shown as the mean ± SEM, n=6. $^{**}P < 0.01$.

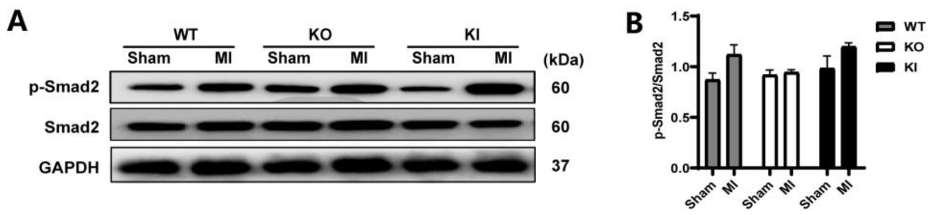

Supplementary Figure S5. The p-Smad2/Smad2 ratio was increased after MI, but the difference was not significant determined by western blot analysis (A) Representative images of the protein levels of p-Smad2 and Smad2. (B) Statistical analysis of the p-Smad2/Smad2 ratio. Data are shown as the mean ± SEM, n=3.
